# Lumbar rachischisis associated with diastematomyelia: prenatal diagnosis and fetopathological examination

**DOI:** 10.11604/pamj.2022.42.146.35644

**Published:** 2022-06-23

**Authors:** Mahdi Farhati, Abir Karoui

**Affiliations:** 1 Obstetrics and Gynecology Department “C”, Tunis Maternity and Neonatology Center, Tunis, Tunisia

**Keywords:** Prenatal diagnosis, rachischisis, spina bifida, diastematomyelia, sonography

## Image in medicine

We report the case of a 29-year-old female patient who presented for a routine ultrasound scan at 17 weeks of gestation. Sonography showed, on an axial view of the fetal brain, flattening of the frontal bones resulting in a lemon like deformity of the cephalic pole (A). Biparietal diameter and head circumference were below 3^rd^ percentile (A). The examination of lumbar spine showed vertebral and skin defect on a sagittal view (B). The diagnosis of Chiari II malformation was suspected. An additional midline echogenic focus (B, C) attracted our attention. Sonography showed widening of the spinal canal in the coronal view (C). Associated diastematomyelia was suspected. Magnetic resonance imaging (MRI) confirmed the presence of lumbar rachischisis associated with diastematomyelia. The couple decided to interrupt the pregnancy. Fetopathological examination confirmed the abnormalities described on ultrasound (A, B, C) and showed an associated ventricular septal defect.

**Figure 1:**
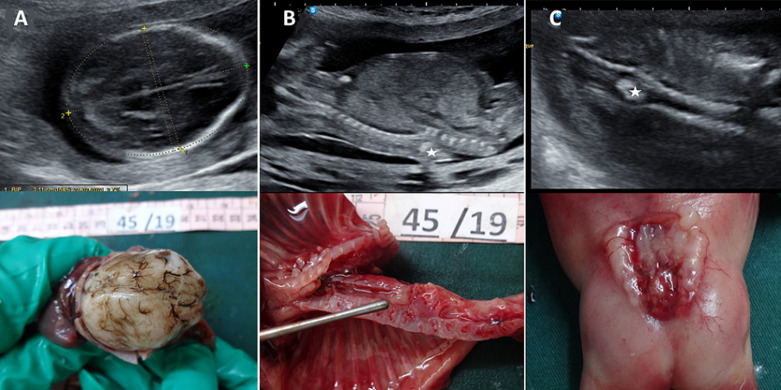
(A, B, C) prenatal sonography findings and fetopathology examination

